# Phosphorus limits *Eucalyptus grandis* seedling growth in an unburnt rain forest soil

**DOI:** 10.3389/fpls.2014.00527

**Published:** 2014-10-06

**Authors:** David Y. P. Tng, David P. Janos, Gregory J. Jordan, Ellen Weber, David M. J. S. Bowman

**Affiliations:** ^1^School of Plant Science, University of TasmaniaHobart, TAS, Australia; ^2^Australian Tropical Herbarium, James Cook UniversityCairns, QLD, Australia; ^3^Department of Biology, University of MiamiCoral Gables, FL, USA; ^4^Wet Tropics Management AuthorityCairns, QLD, Australia

**Keywords:** ashbed effect, *Eucalyptus grandis*, fire ecology, giant eucalypts, phosphorus limitation, seedling growth

## Abstract

Although rain forest is characterized as pyrophobic, pyrophilic giant eucalypts grow as rain forest emergents in both temperate and tropical Australia. In temperate Australia, such eucalypts depend on extensive, infrequent fires to produce conditions suitable for seedling growth. Little is known, however, about constraints on seedlings of tropical giant eucalypts. We tested whether seedlings of *Eucalyptus grandis* experience edaphic constraints similar to their temperate counterparts. We hypothesized that phosphorous addition would alleviate edaphic constraints. We grew seedlings in a factorial experiment combining fumigation (to simulate nutrient release and soil pasteurization by fire), soil type (*E. grandis* forest versus rain forest soil) and phosphorus addition as factors. We found that phosphorus was the principal factor limiting *E. grandis* seedling survival and growth in rain forest soil, and that fumigation enhanced survival of seedlings in both *E. grandis* forest and rain forest soil. We conclude that similar to edaphic constraints on temperate giant eucalypts, mineral nutrient and biotic attributes of a tropical rain forest soil may hamper *E. grandis* seedling establishment. In rain forest soil, *E. grandis* seedlings benefited from conditions akin to a fire-generated ashbed (i.e., an “ashbed effect”).

## INTRODUCTION

Fire is an important phenomenon that influences the dynamics and evolution of many vegetation systems worldwide (Whelan, 1995; Bond et al., 2005; Bowman et al., 2009). In several ecosystems such as some coniferous forests of circumpolar regions (Stocks, 2004; Body et al., 2010), various giant coniferous forests of the Pacific Northwest of the United States (Franklin and Hemstrom, 1981; Agee, 1993), and giant eucalypt forests in temperate Australia (Ashton and Attiwell, 1994; Tng et al., 2012b), regeneration depends primarily on high intensity fires resulting from high fuel loads and rare episodes of severe fire weather. As well as removing dense understories and reducing plant competition, such fires can modify the physical, chemical and biological properties of the soil (Certini, 2005) in ways that promote the early growth of the regenerating forest. This potential growth-promoting effect of fire has been documented for several forest types throughout Australia (Hatch, 1960; Pryor, 1963; Humphreys and Lambert, 1965; Renbuss et al., 1973).

Regeneration promotion by fire–an “ashbed effect”–typically is characterized by an ash-rich germination medium high in plant-available nutrients together with an abundance of safe sites for germination and establishment (Pryor, 1963; Loneragan and Loneragan, 1964; Humphreys and Lambert, 1965). Ash deposited by fire may be high in potassium, calcium, and magnesium, and can raise soil pH in post-fire soils, potentially liberating phosphorus in acid soils. Most importantly, heating soil to 400–600°C, as may be expected of a high-intensity fire, has been shown to increase the availability of phosphorus and nitrogen by killing microbes (Chambers and Attiwell, 1994), in spite of the potential for volatilization loss of nitrogen. Burning established vegetation and litter also potentially increases the number of safe sites by: (i) altering soil physical characteristics, for example, reducing bulk density and thereby increasing water availability (Certini, 2005; Boerner et al., 2009); (ii) denaturing residual plant toxins or inhibitory compounds (Christensen and Muller, 1975; May and Ash, 1990); (iii) removing potentially competing surrounding vegetation (Ashton, 1986); (iv) eliminating other biological opposition to recruitment, such as that by soil-litter microorganisms and pathogens (e.g., Florence and Crocker, 1962; Ellis and Pennington, 1992); and (v) potentially disrupting common mycorrhizal networks (Janos et al., 2013). Elimination of inhibitory soil factors may be especially important as suggested by results from soil of an old-growth *E. regnans* F. Muell. forest that retarded the growth of *E. regnans* seedlings (Ashton and Willis, 1982) and soil from temperate rain forest that inhibited *E. delegatensis* R. T. Baker seedlings (Ellis and Pennington, 1992).

Because temperate giant eucalypt forests typically comprise understories of fire-sensitive rain forest species (Tng et al., 2012b), these forests’ dependence on intense fires and ash beds for eucalypt regeneration is somewhat paradoxical. The association of rain forest species with giant eucalypts such as *E. regnans* in mesic, temperate regions is thought to be a consequence of natural succession (Gilbert, 1959; Jackson, 1968): high intensity fires enable the giant eucalypts to regenerate, after which rain forest species establish as an understory beneath their emergent canopies (Bowman, 2000; Tng et al., 2012b). Somewhat in contrast to this well documented pattern, in the humid tropics of North Queensland, *E. grandis* W. Hill ex Maiden occurs as a canopy dominant within an ecotonal band that separates rain forest from savanna (Unwin, 1989; Harrington et al., 2000). Nevertheless, Bowman (2000) and Tng et al. (2012b) have suggested that tropical *E. grandis* forests may be ecologically similar to temperate giant eucalypt forests in requiring high intensity fires for regeneration (Duff, 1987).

Regeneration of *E. grandis* forests is a matter of considerable current interest because of their uniqueness (Tng et al., 2012b) and because they can contain threatened species of conservation importance. Interest is heightened by concern that *E. grandis* forests might be at risk of being replaced by rain forest. Contemporary trends of rain forest expansion often are at the expense of *E. grandis* forest (Harrington and Sanderson, 1994; Tng et al., 2012a), perhaps because the natural high intensity fires upon which *E. grandis* regeneration depends are rare in the tropics (Little et al., 2012; Tng et al., 2012b). Understanding the regeneration of *E. grandis*, however, currently is hampered by a lack of experimental data on *E. grandis* seedling growth and mineral nutrition in natural settings. In particular, it is not known if ambient tropical rain forest soil limits *E. grandis* seedling growth in the absence of the phosphorus that may be furnished by an ashbed.

The aim of this study was to compare the effects of one tropical rain forest soil versus a nearby *E. grandis* forest soil on the survival and growth of *E. grandis* seedlings. Because we were interested to examine the effects of ambient soils, and because it is not practical to experimentally expose soils to high intensity fire, we used methyl-bromide soil fumigation to simulate partial sterilization and mineral nutrient release by fire without a liming effect of ash or modification of soil structure (Chambers and Attiwell, 1994; Bowman and Fensham, 1995). Because fumigation may eliminate soil pathogens (Ridge, 1976; Ebben et al., 1983) and may release plant available nitrogen and phosphorus (Weston and Attiwell, 1990; Chambers and Attiwell, 1994; Serrasolsas and Khanna, 1995), we predicted that: (i) the survival and growth of *E. grandis* seedlings would be greater in fumigated than in non-fumigated soils, and (ii) poorer growth in rain forest soil than in *E. grandis* forest soil can be overcome by phosphorus fertilization.

## MATERIALS AND METHODS

### SOIL PREPARATION AND ANALYSIS

We collected soil from two forest types, an *E. grandis* forest and an adjacent tropical rain forest at Davies Creek, Far North Queensland (17 1′30′′ S, 145°35′46′′ E) in May, 2010, at the beginning of the dry season. This vicinity also was sampled by Warman et al. (2013) for their comparison of Queensland soils across rain forest boundaries, and their work provides a broad context within which the soils that we used can be placed (**Table 1**).

**Table 1:** Attributes of non-fumigated and fumigated *Eucalyptus grandis* forest and adjacent rain forest soil collected from Davies Creek, Far North Queensland (17 1’30′′ S, 145°35’46′′E).

Attribute (units)	Non-fumigated *E. grandis* forest soil	Fumigated *E. grandis* forest soil	Non-fumigated rain forest soil	Fumigated rain forest soil
Ammonium N (mg kg^-1^)	49 (14.5 ± 3.6)	66	66 (43.3 ± 26.3)	215
Nitrate N (mg kg^-1^)	**14 (b)** (2.4 ± 1.5)	**3 (b)**	**62 (a)** (7.15 ± 5.21)	**61 (a)**
Phosphorus (Colwell) (mg kg^-1^)	9 (13.5 ± 3.1)	10	16 (50.4 ± 48.8)	11
Phosphorus (AEM P_i_) (mg kg^-1^)	6.45	7.17	8.34	4.96
Conductivity (dS m^-1^)	0.103 (0.120 ± 0.016)	0.086	0.227 (0.192 ± 0.08)	0.331
pH in CaCl_2_	**5.2 B, b**	**5.4 A, b**	**5.4 B, a**	**5.6 A, a**
pH in H_2_O	**6.0 B, b** (4.76 ± 0.41)	**6.1 A, b**	**6.1 B, a** (5.67 ± 0. 62)	**6.2 A, a**
Copper (mg kg^-1^)	0.29 (0.54 ± 0.46)	0.82	0.34 (2.08 ± 0.98)	0.46
Iron (mg kg^-1^)	26.9(379 ± 163)	25.8	37.5 (296 ± 144)	41.3
Manganese (mg kg^-1^)	**138 A, a** (13.8 ± 18.5)	**101 B, a**	**78.8 A, b** (75.0 ± 53.3)	**36.3 B, b**
Zinc (mg kg^-1^)	**0.82 (b)** (0.77 ± 0.45)	**0.61 (b)**	**2.44 (a)** (4.46 ± 5.13)	**2.65 (a)**
Exchangeable aluminum (meq 100 g^-1^)	0.118 (3.89 ± 1.21)	0.104	0.077 (1.05 ± 1.45)	0.203
Exchangeable calcium (meq 100 g^-1^)	**7.93 (b)** (1.30 ± 1.41)	**7.27 (b)**	**15.0 (a)** (13.6 ± 15.3)	**15.6 (a)**
Exchangeable magnesium (meq 100 g^-1^)	**2.18 (b)** (1.42 ± 0.35)	**2.11 (b)**	**2.81 (a)** (4.31 ± 3.66)	**2.82 (a)**
Exchangeable potassium (meq 100 g^-1^)	**0.37 (b)** (0.71 ± 0.28)	**0.36 (b)**	**0.73 (a)** (0.85 ± 0.38)	**0.65 (a)**
Exchangeable sodium (meq 100 g^-1^)	0.05 (0.14 ± 0.01)	0.05	0.05 (0.25 ± 0.18)	0.04

*Attributes differing significantly (*P* < 0.075) by two-factor analysis of variance are shown in bold; those followed by different uppercase letters had a significant main effect of fumigation, and those followed by different lowercase letters had a significant main effect of soil type. If significance was marginal (0.05 < *P* < 0.075), the letters are enclosed in parentheses. For comparison, soil attributes (mean ± 1 SD) from Warman et al. (2013) are shown in parentheses beneath the Davies Creek values. Warman et al. (2013) sampled soils from the region; their rain forest means represent five different localities and their wet sclerophyll forest (i.e., *E. grandis* forest) means represent three. AEM *P*_i_ = inorganic phosphorus extracted by anion exchange membrane.*

Although both the *E. grandis* forest and the tropical rain forest where we collected soil were underlain by granite, the two forest types differed drastically in light environment, floristics, and fire risk (Turton and Duff, 1992; Little et al., 2012). The *E. grandis* forest was tall-statured, with an even but open canopy (∼35–50% canopy cover) ranging from 40 to 45 m in height, and with an understory dominated by a mix of grasses together with herbaceous and shrubby rain forest pioneers. The adjacent rain forest was simple notophyll vine forest (Webb, 1959) which principally comprised primary rain forest species. It had a closed canopy (∼75% canopy cover) ranging from 20 to 35 m in height, and was more species rich than the *E. grandis* forest.

Within each forest type, we collected 60 kg of the top 15 cm of soil excluding leaf litter from three places approximately a meter apart and then thoroughly mixed the soil of each forest type separately to homogenize it. The soil was not sieved in order to minimize changes in texture. Half of the soil from each forest type was fumigated with methyl-bromide gas at a rate of 64 g m^-3^ for 24 h in porous sisal bags, each of which was ∼30 cm high when filled and laid on its side. Fumigation by methyl-bromide was chosen because it can release plant-assimilable N and P (Eno and Popenoe, 1964), thereby mimicking that aspect of fire (Bowman and Fensham, 1995) in addition to killing both pathogenic and beneficial microbes. The soil was aired inside a shelter for 1 week before use.

To determine the mineral nutrient content of each soil both before and after fumigation, samples were sent for analysis to a commercial soil laboratory in Western Australia. There, ammonium and nitrate were extracted in KCl; P (Colwell) in sodium bicarbonate; soil pH and electrical conductivity were determined in a 1:5 soil:water suspension; Cu, Fe, Mn, and Zn in a DTPA extract; and Ca, Mg, K, and Na in an ammonium chloride extract. Additional samples of each soil were sent to the School of Plant Biology, University of Western Australia for analyses of plant-available inorganic phosphorus (P_i_) by anion exchange membrane (AEM) extraction (Nuernberg et al., 1998).

To compare the effect of fumigation with that of burning, we opportunistically collected ambient and burnt soil for AEM P_i_ analysis from another *E. grandis* forest near Ravenshoe, Queensland (17 39′24′′ S, 145°30′35′′ E) that is physiognomically similar to the Davies Creek forest, but which was affected by a wildfire in September, 2012. At the Ravenshoe site, we collected a total of three kilograms of the top 15 cm of soil excluding leaf litter from three places approximately a meter apart and then thoroughly mixed the soil from burned and unburnt *E. grandis* forest separately to homogenize it.

### SEEDLING GROWTH

*Eucalyptus grandis* seeds from naturally occurring populations in North Queensland were not available for the experiment, so we obtained seeds from a plantation south of Grafton, on the east coast of New South Wales, Australia. Seeds were sown onto a shallow tray containing fumigated *E. grandis* forest soil on May 16, 2011. Germination occurred within a week. Two weeks after germination, seedlings were transplanted (one per pot) into 10 cm diameter pots containing 600 cm^3^ of soil. Care was taken to ensure that seedling root systems were not damaged during transplant. Initially, four treatments of 60 plants each were established: fumigated *E. grandis* forest soil; non-fumigated *E. grandis* forest soil; fumigated rain forest soil; and non-fumigated rain forest soil. Subsequently, half of the surviving plants in each treatment were fertilized with phosphorus to constitute a fully crossed, 3 factor experiment with soil type (rain forest versus *E. grandis* forest soil), fumigation (or not), and P addition (or not) as factors.

At 86 days after transplant (DAT), we began P addition. In order to equalize seedling sizes between P-fertilized and not fertilized groups within treatments, the surviving plants from each initial soil type × fumigation treatment were rank-ordered by height, and every second seedling was assigned to be fertilized weekly with 15 ml of 0.8 mg P ml^-1^. The P solution was prepared by diluting triple superphosphate [i.e., monocalcium phosphate monohydrate, Ca(H_2_PO_4_)_2_ ⋅ H_2_O] in water. No other fertilizer was added.

After transplant, pots were arranged on a metal rack supported 70 cm above ground under ambient outdoor conditions in Cairns, QLD, Australia (16 51′03′′ S, 145°44′53′′ E), and were watered daily during the dry season with tap water as needed to maintain the soil near field capacity. Pots containing fumigated and non-fumigated soils were arranged in separate blocks 100 cm apart to minimize potential movement of arbuscular mycorrhizal fungus spores between treatments by water splash. No microbial filtrate (Koide and Li, 1989; Allen et al., 1993) was added to any of the soils. The pots were rearranged every 3 weeks to mitigate position effects. Weeds and invertebrate herbivores were removed manually upon detection. Native weeds were found only in pots of non-fumigated soil, attesting to successful fumigation.

Beginning immediately after transplant, we measured seedling height from the soil surface to the shoot apex, censused mortality, and also tabulated mineral nutrient deficiency symptoms based on leaf color (Dell, 1996) at irregular, 20–33 d intervals. Final growth measurements and leaf color assessments were made at 146 DAT when the experiment was harvested. We harvested the experiment at that time because 13 seedlings exhibited symptoms of damping off or abnormal leaf development. All aboveground shoot tissues (including all stems and leaves) were harvested, dried in an oven at 60°C for 1 week and weighed. After weighing, the shoots were ground to powder and analyzed for total nitrogen and phosphorus at the School of Plant Biology, University of Western Australia. At least 0.2 g ground tissue was needed, but because many small plants provided insufficient tissue individually, we combined plants within treatments by quantiles of seedlings rank-ordered by height within each treatment. This resulted in three composite samples from the non-fertilized, fumigated rain forest soil treatment, five from the non-fertilized, non-fumigated rain forest soil treatment, and eight from each of the other six treatments (in which the largest plants were found). The 13 seedlings that exhibited pronounced symptoms of disease were excluded from the analyses. Those 13 seedlings were distributed relatively evenly among the eight treatments and therefore their exclusion was not likely to bias comparisons among treatments.

### MYCORRHIZA ASSESSMENT

After harvesting aboveground shoot tissues, we extracted the fine roots of six randomly selected plants per treatment by gentle rinsing over a 2 mm sieve, and we preserved the roots in 50% ethanol for later assessment of mycorrhizas. Subsequently, the preserved roots were cleared in 10% KOH at room temperature for 48 h and then stained in 0.05% trypan blue in lactoglycerol. For each plant sample, we mounted ten, 1–2 cm root pieces including lateral, ultimate rootlets on microscope slides and checked for the presence of ectomycorrhizas or, in their absence, arbuscular mycorrhizas (typical hyphae and vesicles in the root cortex) with a compound microscope at 200-times magnification. We used the magnified gridline intersection method (McGonigle et al., 1990) with assessment of 100 intersections per seedling to quantify mycorrhizas.

### DATA ANALYSIS

Because we submitted only single samples of our homogenized, non-fumigated and fumigated *E. grandis* forest and rain forest soils for physicochemical analyses, we compared them by two-way analyses of variance (ANOVA) using the interaction term as the error estimate. Because of the limited power of these analyses, we did not Bonferroni-correct for the number of soil parameters examined.

The onset of P addition at 86 DAT divided our experiment into two time segments that we analyzed separately. The effects of soil type and fumigation were analyzed from seedling transplant to 86 DAT, and the effects of soil type, fumigation and P addition were analyzed from 86 DAT to harvest at 146 DAT. To test for differences in survival and foliar P-deficiency symptoms among treatments, we used two-way, factorial logistic regressions against soil type and fumigation for the initial 86 DAT, and three-way, factorial logistic regressions with soil type, fumigation and P addition as factors from 86 to 146 DAT. For the survival analysis, we used all seedlings that were surviving, including those that were diseased. For foliar P-deficiency symptoms, 209 surviving seedlings at 86 DAT and 193 surviving and non-diseased seedlings at 146 DAT were analyzed. For the 146 DAT analyses, we used the number of seedlings among those 193 seedlings which had changed from markedly purple to green as the response variable. These logistic regression results are reported as χ^2^ values and their associated probabilities.

To analyze the effects of soil type, fumigation and their interaction prior to fertilization on seedling height, we analyzed height at 86 DAT with a two-way ANOVA by using the aov function of R (Version 2.7.1, R Development Core Team, 2004). For growth from 86 to 146 DAT, we performed a three-way ANOVA on a log-transformed height ratio that was obtained by dividing the seedling height at 146 DAT by that at 86 DAT. Tukey’s honestly significant difference (HSD) tests with *P* ≤ 0.05 were used to identify differences among treatments.

For harvest data, the effects of soil type, fumigation, P addition and their interactions on aboveground shoot dry weight, and aboveground shoot N and P concentrations were analyzed by three-way ANOVA. Tukey’s HSD tests with *P* ≤ 0.05 were used to compare treatment means. For the dry weight analyses there were 193 seedlings, but for the N and P concentrations 56 composited quantiles represented those 193 seedlings.

## RESULTS

### SOIL ANALYSES

We detected no significant differences between *E. grandis* forest soil and rain forest soil, whether fumigated or not, for ammonium, Colwell P, AEM P_i_, conductivity, extractable Cu, Fe, exchangeable Al or Na (**Table 1**). In spite of the limited power of our statistical analyses, however, Mn was significantly higher in *E. grandis* forest soil than in rain forest soil, but was significantly diminished by fumigation. In contrast, both measures of pH were significantly higher for rain forest soil than for *E. grandis* forest soil, and fumigation elevated pH in both soils. Although not affected significantly by fumigation, exchangeable Mg and marginally significantly (*F*_1,3_ = 137.3, *P* = 0.0542) exchangeable Ca were higher in rain forest than in *E. grandis* forest soil, consistent with the pH difference between the soils. Also marginally significantly higher in rain forest than in *E. grandis* forest soil were nitrate (*F*_1,3_ = 112.36, *P* = 0.0599), Zn (*F*_1,3_ = 75.94, *P* = 0.0727), and exchangeable K (*F*_1,3_ = 86.22, *P* = 0.0683). *E. grandis* forest soil at Ravenshoe that was affected by a wildfire had 10.9 mg kg^-1^ AEM P_i_, while unburnt soil had 7.0 mg kg^-1^.

### MORTALITY AND FOLIAR PHOSPHORUS DEFICIENCY SYMPTOMS

**FIGURE 1:**
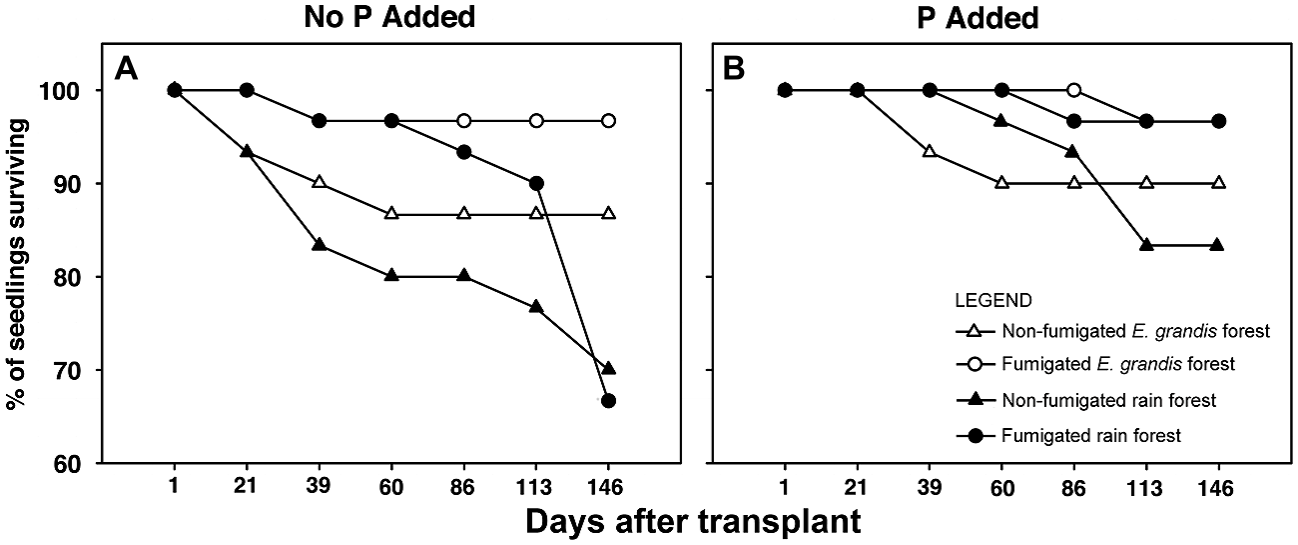
**Percentage of *Eucalyptus grandis* seedlings surviving when grown with no phosphorus (P) added **(A)** or with phosphorus added **(B)** versus days after transplant (DAT).** P addition began 86 DAT. Percentage survival is based on 30 replicate plants in each treatment.

By 86 DAT, mortality of seedlings differed significantly between fumigated and non-fumigated treatments (χ^2^ = 7.91, *P* = 0.0049) with seedlings in non-fumigated soils of both types having significantly lower survival than those grown in fumigated soil (**Figure 1**). Between 86 and 146 DAT, fumigation alone ceased to have a significant effect (χ^2^ = 0.19, *P* = 0.667), but soil type (χ^2^ = 8.215, *P* = 0.0042), soil type × P addition (χ^2^ = 4.47, *P* = 0.034), and fumigation × P addition (χ^2^ = 4.63, *P* = 0.031) significantly affected survival. Overall, there was lower survival in non-fertilized fumigated and non-fumigated rain forest soils than in any other treatment (**Figure 1**). All treatments receiving P addition had higher survival percentages than their non-fertilized counterparts (**Figure 1**).

**FIGURE 2:**
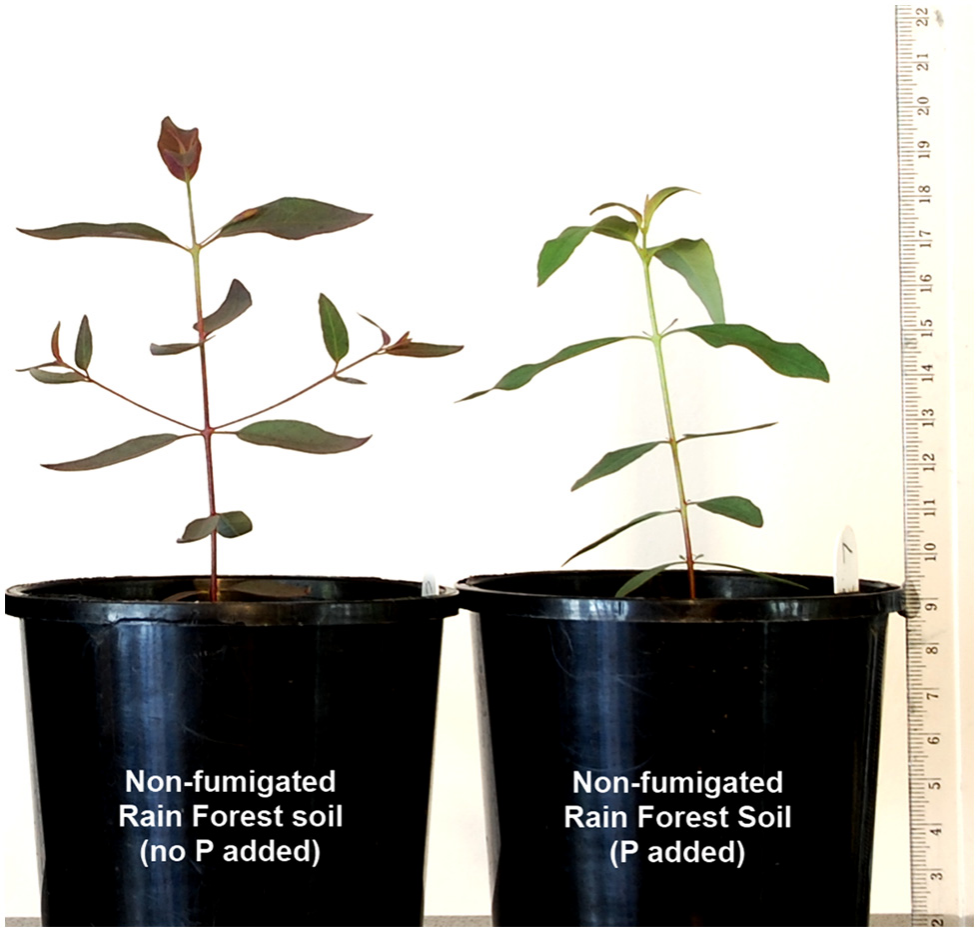
**Examples of *Eucalyptus grandis* seedlings grown for 146 days after transplant (DAT) to non-fumigated rain forest soil without or with phosphorus (P) addition.** P deficiency in the non-fertilized seedling **(left)** is indicated by conspicuous purple leaf coloration (see Dell, 1996) in comparison to the green leaves of the P-fertilized plant **(right)**.

**FIGURE 3:**
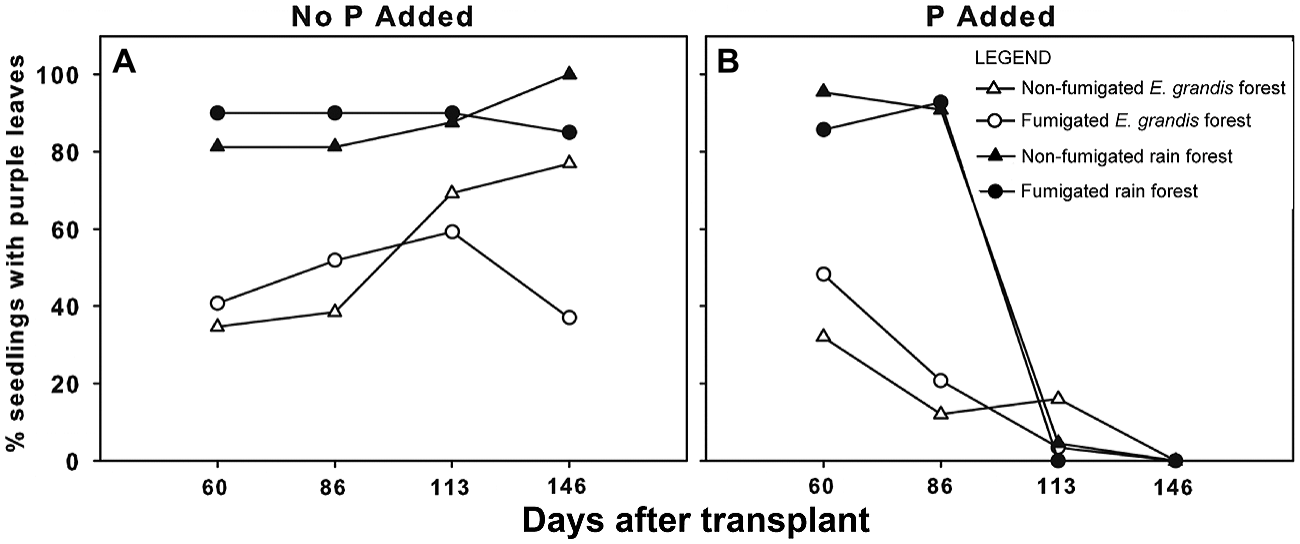
**Percentage of *Eucalyptus grandis* seedlings exhibiting purple leaf coloration (indicative of phosphorus deficiency; see **Figure 2**) when grown with no phosphorus (P) added **(A)** or with phosphorus added **(B)** versus days after transplant (DAT).** P addition began 86 DAT. The numbers of surviving seedlings at 146 DAT were: non-fumigated *E. grandis* (*n* = 26 no P added, 25 P added), Fumigated *E. grandis* (*n* = 27 no P added, 29 P added), Non-fumigated rain forest (*n* = 16 no P added, 22 P added) and Fumigated rain forest (*n* = 20 no P added, 28 P added). Thirteen seedlings which were diseased at 146 DAT were excluded.

By 86 DAT, some seedlings of all treatments showed purple coloration of leaves (**Figure 2**) symptomatic of phosphorus deficiency. Other deficiency symptoms were not apparent. To 86 DAT, the percentage of seedlings with purple leaves was higher for seedlings in rain forest soil than for those in *E. grandis* forest soil (χ^2^ = 120.2, *P* < 0.0001) and also was higher for those in fumigated soils than in non-fumigated soils (χ^2^ = 6.27, *P* = 0.0123; **Figure 3**). At 146 DAT, fumigation (χ^2^ = 7.73, *P* = 0.0054), P addition (χ^2^ = 41.39, *P* < 0.0001), and fumigation × P addition (χ^2^ = 5.28, *P* = 0.022) significantly affected the percentages of P-deficient seedlings. There was a steep decline in the percentage of P-deficient seedlings in all treatments with P addition, and by the end of the study, none of the fertilized seedlings showed symptoms of P deficiency (**Figure 3**). In contrast, when not fertilized, the percentage of P-deficient seedlings in both the non-fumigated *E. grandis* forest soil and the non-fumigated rain forest soil treatments continued to increase over the course of the experiment, and in the latter treatment, all surviving seedlings were P-deficient by 146 DAT (**Figure 3**).

### GROWTH ATTRIBUTES, SHOOT N AND P CONCENTRATIONS, AND MYCORRHIZAL COLONIZATION

**FIGURE 4:**
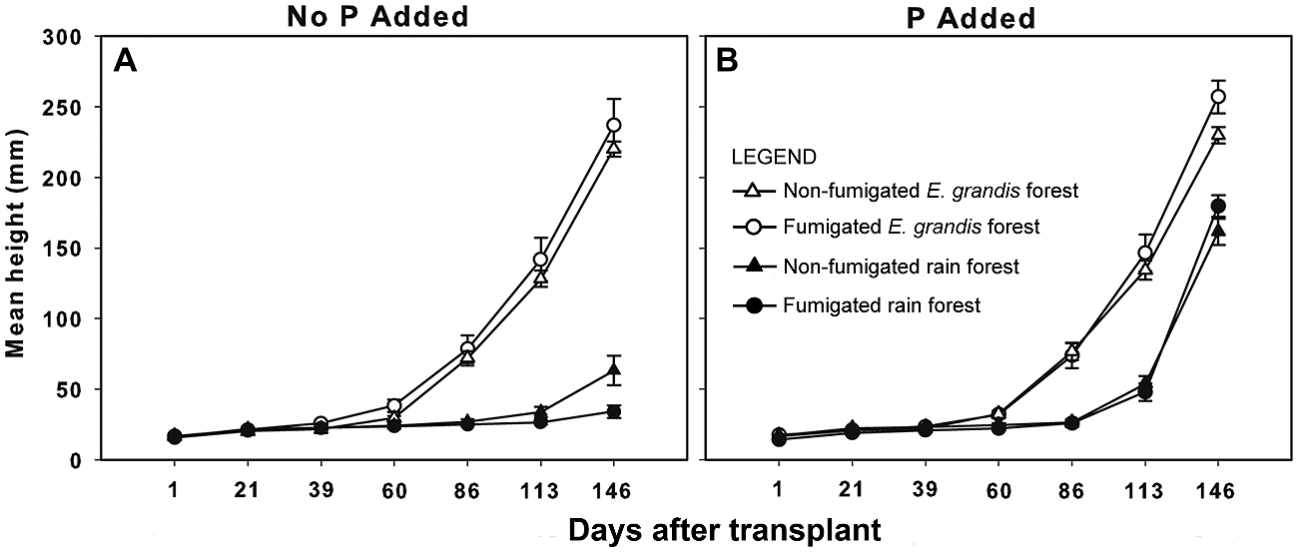
**Mean height (mm ± SE) of *Eucalyptus grandis* seedlings grown with no phosphorus (P) added **(A)** or with phosphorus added **(B)** versus days after transplant (DAT).** P addition began 86 DAT. The numbers of surviving replicates at 146 DAT were: non-fumigated *E. grandis* (*n* = 26 no P added, 25 P added), Fumigated *E. grandis* (*n* = 27 no P added, 29 P added), Non-fumigated rain forest (*n* = 16 no P added, 22 P added) and Fumigated rain forest (*n* = 20 no P added, 28 P added). Thirteen seedlings which were diseased at 146 DAT were excluded.

Immediately before fertilization at 86 DAT, seedlings grown in *E. grandis* forest soil regardless of fumigation on average were almost three times taller than those grown in rain forest soil (**Figure 4**). A two-way ANOVA confirmed that this difference was significant (*F*_1,216_ = 236, *P* < 0.0001). The ANOVA detected no significant effect of fumigation (*F*_1,216_ = 1.88, *P* = 0.18), however, nor any interaction between fumigation and soil type (*F*_1,216_ = 0.22, *P* = 0.63).

After P addition commenced, the log-transformed height ratio analyzed by three-way ANOVA was significantly affected by soil type, P addition, soil type × fumigation, soil type × P addition, and fumigation × P addition (**Table 2**). Tukey’s HSD tests showed significant differences between fertilized rain forest seedlings and those not fertilized, and also between all groups of seedlings in rain forest soil and those in *E. grandis* forest soil. Seedlings in *E. grandis* forest soil consistently were the tallest (**Figure 4**).

**Table 2:** Three-way analysis of variance results for the effects of soil type, fumigation, and phosphorus addition on *Eucalyptus grandis* seedling attributes 146 days after transplant (DAT).

Seedling attribute	Height ratio	Shoot dry weight	Shoot N concentration	Shoot P concentration
Factor or interaction	*F (P)*	*F (P)*	*F (P)*	*F (P)*
Soil	**8.81 (<0.0034)**	**141 (<0.0001)**	**33 (<0.0001)**	**17 (0.0002)**
Fumigation	2.51 (0.115)	3.35 (0.069)	2.83 (0.099)	**5.94 (0.0185)**
P addition	**225 (<0.0001)**	**93.4 (<0.0001)**	**11 (0.002)**	**292 (<0.0001)**
Soil × fumigation	**4.86 (0.029)**	0.19 (0.662)	**17.4 (0.0001)**	1.38 (0.246)
Soil × P addition	**181 (<0.0001)**	**81.5 (<0.0001)**	**12.5 (0.0009)**	**39 (<0.0001)**
Fumigation × P addition	**7.63 (0.0063)**	0.2 (0.654)	2.15 (0.149)	**4.22 (0.045)**
Soil × fumigation × P addition	2.24 (0.137)	0.004 (0.951)	1.52 (0.223)	0.64 (0.429)

**FIGURE 5:**
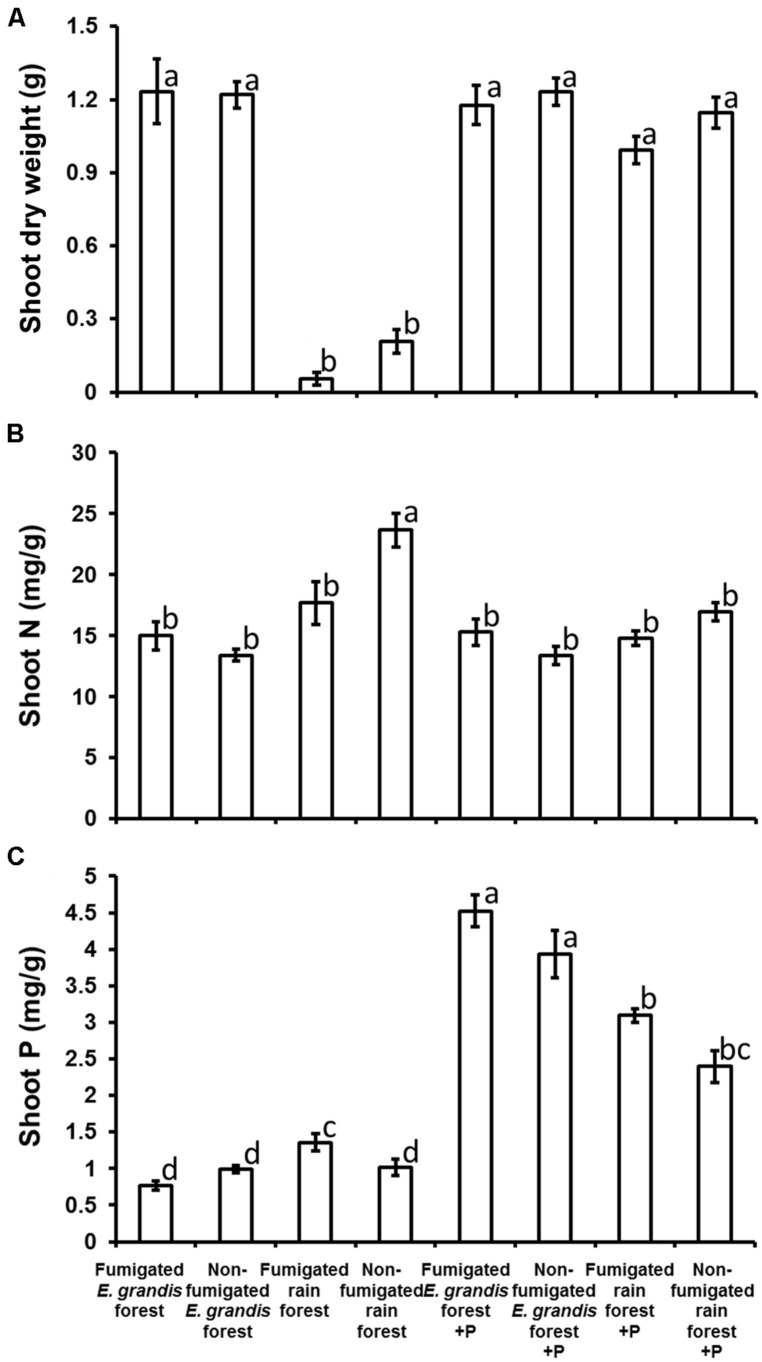
**Soil type (*E. grandis* forest versus rain forest), fumigation, and P addition (+P) effects on *Eucalyptus grandis* seedling (mean ± SE) shoot dry weight (g; **A**), shoot nitrogen concentration (mg/g; **B**), and shoot phosphorus concentration (mg/g; **C**) at harvest, 146 days after transplant (DAT).** Bars topped by the same lowercase letter do not differ significantly at *P* ≤ 0.05 by Tukey’s honestly significant difference *post hoc* test. The numbers of surviving replicates at 146 DAT were: non-fumigated *E. grandis* (*n* = 26 no P added, 25 P added), Fumigated *E. grandis* (*n* = 27 no P added, 29 P added), Non-fumigated rain forest (*n* = 16 no P added, 22 P added) and Fumigated rain forest (*n* = 20 no P added, 28 P added). Thirteen seedlings which were diseased at 146 DAT were excluded.

*
The attributes are the log-transformed ratio of height 146 DAT to height 86 DAT, aboveground dry weight at harvest at 146 DAT, and shoot tissue N and P concentrations determined from composited plant tissues. Statistically significant effects (P . 0.05) are shown in bold. See **Figures 4 and 5** for treatment means. Numerator degrees of freedom are 1 for all factors and interactions, and denominator, error degrees of freedom are 171, 186, 48, and 48 for height ratio, shoot dry weight, and shoot N and P concentrations, respectively.*

Mean aboveground dry weights of *E. grandis* seedlings at 146 DAT were affected significantly by soil type, P addition, and soil type × P addition, but neither fumigation nor any of its interactions affected dry weight significantly (**Table 2**). Seedlings grown in rain forest soil without P addition had significantly lower mean aboveground dry weights than those in any other treatment (**Figure 5A**).

Mean aboveground shoot N concentrations of *E. grandis* seedlings at 146 DAT were affected significantly by soil type, P addition, soil type × fumigation, and soil type × P addition (**Table 2**). Seedlings of the non-fumigated, non-fertilized rain forest soil had significantly higher aboveground shoot N concentrations than those of any other treatment (**Figure 5B**). In contrast to N, mean aboveground shoot P concentrations were affected significantly by all main factors (soil type, fumigation, and P addition) as well as by soil type × P addition, and fumigation × P addition interactions (**Table 2**). When seedlings were not fertilized, only those in fumigated rain forest soil had an elevated mean shoot P concentration, but when fertilized, seedlings in *E. grandis* forest soil had the highest aboveground shoot P concentrations of all, and they significantly exceeded those of seedlings in rain forest soil which tended to be higher than those of seedlings in most non-fertilized treatments (**Figure 5C**).

Although we examined at least 10 cm root length for each of 48 seedlings (six seedlings per treatment × eight treatments) for a total length examined of approximately 5 m, we found no fully formed ectomycorrhizas, nor did we find arbuscular mycorrhizal fungus colonization at any of the 4800 examined gridline intersections with roots. Away from gridline intersections, however, we very rarely did see typical glomeromycotan hyphae as well as a few vesicles in root cortices. Although we did not quantify them, septate hyphae were relatively common in seedlings of all treatments. Some septate hyphae had clamp connections and may have been incipient ectomycorrhizal colonization, but most lacked clamps. Sometimes septate, melanized hyphae were accompanied by microsclerotia suggestive of “dark septate endophytes” (Mandyam and Jumpponen, 2005). We also found endobiotic, holocarpic chytrid sporangia in *E. grandis* seedling fine roots.

## DISCUSSION

We simulated aspects of *E. grandis* regeneration by studying seedling growth in fumigated versus non-fumigated *E. grandis* forest and adjacent rain forest soils as well as response to P addition in those soils. Although some eucalypts such as *E. obliqua* have distinct ecotypes (Bloomfield et al., 2011), ecotypic differences are not known for *E. grandis* (Jones et al., 2006). High survival and vigorous growth of seedlings in our non-fumigated *E. grandis* forest soil suggests that the New South Wales provenance of our seeds did not constrain our results.

Our non-fumigated rain forest soil fell within the ranges (means ± 1 SD) of parameters reported by Warman et al. (2013) for five Queensland rain forests, except for nitrate (8.7 times their reported mean), Cu (two-tenths of theirs), Fe (one-tenth of theirs) and exchangeable Na (two-tenths of theirs; **Table 1**). Hence, especially with respect to N nutrition, our rain forest soil might have been slightly more favorable for seedling growth than the average Queensland rain forest soil. In contrast, our non-fumigated *E. grandis* forest soil differed considerably from the three wet sclerophyll forests (i.e., *E. grandis* forests) studied by Warman et al. (2013) by having 3.4 times the ammonium, 5.9 times the nitrate, 10 times the Mn, 1.5 times the exchangeable Mg, slightly higher pH, and lower exchangeable Al, K, Na, and conductivity. Nevertheless, those differing parameters of our *E. grandis* forest soil often were closer to values reported by Warman et al. (2013) for rain forest soils than to those for wet sclerophyll forests. Thus, our *E. grandis* forest soil might have been more favorable nutritionally for *E. grandis* seedling growth than wet sclerophyll forest soils generally.

For both of the soils that we studied, even though the only significant effect of fumigation on soil chemistry was to diminish extractable Mn, fumigation enhanced early seedling survival (to 86 DAT) and reduced foliar symptoms of P deficiency throughout our 146 day experiment. In spite of more favorable chemical attributes of rain forest than *E. grandis* forest soil, without fumigation, seedlings survived poorly in rain forest soil–100% showed symptoms of P deficiency at harvest–and they had slower height increase and lower mean shoot dry weight than seedlings in *E. grandis* forest soil. Phosphorus addition to rain forest soil, however, improved seedling survival, completely eliminated P deficiency symptoms, produced high rates of height increase and similar shoot dry weights to seedlings in *E. grandis* forest soil.

These results accord with our predictions and suggest that as for *E. regnans* (Chambers and Attiwell, 1994), release of assimilable phosphorus by a high-intensity fire event may be necessary for *E. grandis* regeneration on rain forest soil. Our results also are consistent with soil fumigation, like intense fires, potentially alleviating inhibitory effects of soil microbiota on seedling survival. Although fire may volatilize some N and S, intense fires kill microbes and thereby release microbially sequestered N and P in addition to diminishing the P-immobilization capacity of soil (Weston and Attiwell, 1990; Chambers and Attiwell, 1994; Serrasolsas and Khanna, 1995). Release of assimilable N and P by fire is corroborated by wood cores of adult *E. grandis* having peaks of N and P that correspond to episodes of fire activity (Heinrich, 2006). We found AEM P_i_ in *E. grandis* forest soil at Ravenshoe, Queensland was elevated by a burn. Unlike intense fires, however, in our experiment fumigation did not significantly alter ammonium, nitrate, Colwell P, or AEM P_i_ of our soils, so its principal influence most likely was to have diminished soil microbial inhibition of *E. grandis* seedlings.

In spite of our inability to detect differences in P concentrations between soil types or in consequence of fumigation, our study strongly underscores the importance of phosphorus in the mineral nutrition of *E. grandis* seedlings as for other eucalypt species (Dell et al., 1987). Similar to our results, other investigators also have shown that P addition stimulated *E. grandis* seedling growth (Mulligan and Sands, 1988; Kirschbaum et al., 1992). In our experiment, even though seedling aboveground dry weight was not affected by P addition to *E. grandis* forest soil, P addition did increase aboveground shoot P concentrations significantly in *E. grandis* soil as well as in rain forest soil. High seedling P concentrations with little growth response suggest luxury accumulation of P (De Mazancourt and Schwartz, 2012) and suggest that growth was limited by another mineral nutrient in P-fertilized *E. grandis* forest soil.

It is somewhat perplexing that when not fertilized, even though seedling shoot P concentrations differed little between soil types, seedlings had higher aboveground dry weight in *E. grandis* soil than in rain forest soil. Little difference in shoot P concentrations, however, is consistent with similar Colwell P and AEM P_i_ in both soil types. The only mineral nutrient conspicuously more abundant in *E. grandis* forest soil than in rain forest soil was Mn, and because Mn strongly influences electron transport and photosynthesis (Raven et al., 2005), we hypothesize that high Mn availability in *E. grandis* forest soil may have contributed to greater seedling height growth than in rain forest soil when neither was P-fertilized. Eucalypts can accumulate very high Mn in their foliage, but how much is necessary is uncertain (Hill et al., 2001). That Mulligan (1989) found no effect of a very low P supply on rate of net photosynthesis by *E. grandis* seedlings bolsters our interpretation. Because our soil types were similar in conductivity, exchangeable Al, and exchangeable Na, it is unlikely that mineral salts were inhibitory in rain forest soil. The effect of Mn that we hypothesize might be peculiar to our Davies Creek site, however, because Warman et al. (2013) found Mn availability generally to be higher in rain forest than in *E. grandis* forest soil. Moreover, in our experiment, fumigation diminished extractable Mn in both soil types, but increased survival and diminished P deficiency symptoms suggesting that there may have been a slight, but analytically non-detectable increase of soil P with fumigation.

In our experiment, although nitrate and exchangeable K were marginally more abundant in rain forest than in *E. grandis* forest soil, neither had any evident effect on *E. grandis* seedling performance (and neither did pH, Mg, Ca, or Zn which also tended to be higher in rain forest than in *E. grandis* forest soil). High seedling aboveground shoot N concentrations were associated with the lowest seedling dry weights because mean N concentration was significantly highest in non-fertilized, non-fumigated rain forest soil and second highest (non-significantly) in non-fertilized, fumigated rain forest soil. Consequently, the low shoot N concentrations of other treatments with statistically indistinguishable, high aboveground dry weights probably reflect a dilution effect (Johnson et al., 1980) of plant size. Mulligan and Sands (1988) similarly found high foliar N concentrations in *E. grandis* grown under phosphorus limitation and suggested that poor growth might have been exacerbated by accumulation of N causing ion imbalance and N toxicity.

While methyl-bromide fumigation of soil may not elevate available mineral nutrients to the same extent as heating of soil (Eno and Popenoe, 1964) or an actual fire, in our experiment, fumigation improved seedling survival in both soil types which might have reflected a reduction of parasitic and pathogenic microorganisms. In unburnt, temperate *E. regnans* forest for example, the fungus *Cylindrocarpon destructans* (Zinssm.) Scholten is a common pathogen that can affect seedling growth negatively (Ashton and Willis, 1982; Iles et al., 2010). Bowman and Fensham (1995) found for the tropical eucalypt *E. tetrodonta* F. Muell. that non-fumigated monsoon rain forest soil significantly inhibited seedling growth and that the inhibition was not alleviated by an NPK fertilizer. In our experiment at harvest, chytrids, and dark septate endophytes were relatively common in *E. grandis* seedling roots, and tree species tend to respond negatively to such endophytes (Mayerhofer et al., 2013).

Besides reducing parasitic and pathogenic microorganisms, fumigation also may diminish or alter mycorrhizal fungus populations. In our seedlings in both fumigated and non-fumigated soils, however, we found no fully intact ectomycorrhizas and very little root colonization by arbuscular mycorrhizal fungi (none at our censused root gridline intercepts). That finding strongly suggests that *E. grandis* seedlings are highly facultatively mycotrophic (sensu Janos, 2007), well able to grow in the absence of any mycorrhizas provided that adequate P is available. In accord with this inference, Pagano and Scotti (2008) concluded from an examination of mycorrhizas of plantation-grown trees that *E. grandis* is not dependent on arbuscular mycorrhizas for growth. Moreover, arbuscular mycorrhizal fungi can affect *E. grandis* seedlings negatively, and such an effect might have contributed to the beneficial effects of fumigation that we observed. For example, Lapeyrie et al. (1992) stated that although arbuscular mycorrhizal inoculation had no effect versus non-inoculated *E. grandis* seedlings, when combined with ectomycorrhizal inoculation, arbuscular mycorrhizas had a negative effect versus solely ectomycorrhizal plants. Fernandes et al. (1999) similarly reported that inoculation of *E. grandis* seedlings with an arbuscular mycorrhizal fungus alone had no effect, but that dual inoculation reduced ectomycorrhiza formation. In a study of another tropical Australian eucalypt, *E. tetrodonta*, Janos et al. (2013) found that common arbuscular mycorrhizal networks exacerbated belowground competition between *E. tetrodonta* seedlings and an exclusively arbuscular mycorrhizal rain forest tree species, and they concluded that fire-caused mortality of rain forest plants likely disrupted inimical arbuscular mycorrhizal fungus networks.

Overall, that survival of *E. grandis* seedlings was significantly improved by fumigation of both *E. grandis* and rain forest soils, that P deficiency symptoms were less and seedling growth was greater in *E. grandis* forest soil than in rain forest soil, and that P addition to rain forest soil alleviated deficiency symptoms and improved seedling survival and growth indicate that *E. grandis* seedling establishment in rain forest soils may be facilitated by high-intensity fires that increase phosphorus availability and diminish rain forest soil microbial inhibition. Phosphorus has been shown similarly to be the primary mineral nutrient limiting regeneration of the temperate, giant eucalypt *E. regnans* in unburnt soils (Ashton and Martin, 1982; Ashton and Kelliher, 1996), suggesting concordant regeneration niches for *E. grandis* and *E. regnans* (Tng et al., 2012b).

## Conflict of Interest Statement

The authors declare that the research was conducted in the absence of any commercial or financial relationships that could be construed as a potential conflict of interest.
